# Manual therapy ameliorates delayed-onset muscle soreness and alters muscle metabolites in rats

**DOI:** 10.14814/phy2.12279

**Published:** 2015-02-23

**Authors:** Susumu Urakawa, Kouichi Takamoto, Tomoya Nakamura, Shigekazu Sakai, Teru Matsuda, Toru Taguchi, Kazue Mizumura, Taketoshi Ono, Hisao Nishijo

**Affiliations:** 1 Department of Judo Neurophysiotherapy, Graduate School of Medicine and Pharmaceutical Sciences, University of ToyamaToyama, Japan; 2 Department of System Emotional Science, Graduate School of Medicine and Pharmaceutical Sciences, University of ToyamaToyama, Japan; 3 Department of Physical Therapy, College of Life and Health Sciences, Chubu UniversityKasugai, Japan; 4 Department of Neuroscience II, Research Institute of Environmental Medicine, Nagoya UniversityChikusa-ku, Nagoya, Japan

**Keywords:** Branched-chain amino acids, pain, eccentric contraction, massage, mechanical hyperalgesia, metabolomics

## Abstract

Delayed-onset muscle soreness (DOMS) can be induced by lengthening contraction (LC); it can be characterized by tenderness and movement-related pain in the exercised muscle. Manual therapy (MT), including compression of exercised muscles, is widely used as physical rehabilitation to reduce pain and promote functional recovery. Although MT is beneficial for reducing musculoskeletal pain (i.e. DOMS), the physiological mechanisms of MT remain unclear. In the present study, we first developed an animal model of MT in DOMS; LC was applied to the rat gastrocnemius muscle under anesthesia, which induced mechanical hyperalgesia 2–4 days after LC. MT (manual compression) ameliorated mechanical hyperalgesia. Then, we used capillary electrophoresis time-of-flight mass spectroscopy (CE-TOFMS) to investigate early effects of MT on the metabolite profiles of the muscle experiencing DOMS. The rats were divided into the following three groups; (1) normal controls, (2) rats with LC application (LC group), and (3) rats undergoing MT after LC (LC + MT group). According to the CE-TOFMS analysis, a total of 171 metabolites were detected among the three groups, and 19 of these metabolites were significant among the groups. Furthermore, the concentrations of eight metabolites, including branched-chain amino acids, carnitine, and malic acid, were significantly different between the LC + MT and LC groups. The results suggest that MT significantly altered metabolite profiles in DOMS. According to our findings and previous data regarding metabolites in mitochondrial metabolism, the ameliorative effects of MT might be mediated partly through alterations in metabolites associated with mitochondrial respiration.

## Introduction

Manual therapy (MT), such as massage, is a type of complementary and alternative medical treatment that has been widely accepted as effective especially for musculoskeletal pain (Barnes et al. 2008; Nelson 2013). Excessive muscle use (i.e. overwork) or unaccustomed exercise usually leads to muscle pain expressed discomfort or soreness, called delayed-onset muscle soreness (DOMS) (Armstrong 1984). DOMS can be specifically induced by lengthening contractions (LC; contraction during muscle stretch, often called “eccentric contraction”); it is characterized by tenderness and movement-related pain in the exercised muscle. MT is recognized as a therapeutic intervention for DOMS in the field of sports (Cheung et al. 2003; Zainuddin et al. 2005). Furthermore, symptoms of DOMS may have underlying etiology in common with painful trigger points, as there are a great deal of similarities in symptoms between the two (Mizumura et al. 2010). Compression at trigger points is an effective MT technique for treating myofascial pain syndrome (Simons 1984; Simons et al. 1999).

However, the objective effects of MT should be identified, although most reports of MT in DOMS have focused on subjective pain in human subjects (Nelson 2013). Recently, Crane et al. (2012) has reported the cellular and molecular alterations due to MT after exercise by using muscle biopsies from human subjects. Although they used muscle exercise by using ergometer without LC instead of the DOMS model, they revealed that MT (effleurage, petrissage, and stripping) 10 min after exercise not only attenuated inflammatory signaling (i.e. NF*κ*B, IL-6, and TNF-*α*), but also promoted mitochondrial functions (i.e. upregulation of mitochondrial electron transport chain components COX7B and ND1). According to these findings, inflammatory signals and metabolites associated with mitochondrial respiration during an early phase might be related to the objective effects of MT. It has been suggested that metabolic stress in an early phase after exercise can induce DOMS after a few days (Braun and Dutto 2003; Tee et al. 2007). Furthermore, soreness usually appears between 8 and 24 h after LC, and peaks at 24 to 48 h (Fridén 1984; Ebbeling and Clarkson 1989; Cleak and Eston 1992). Therefore, the ameliorative effects of MT on DOMS might be attributed to the promotion of metabolic recovery in mitochondria especially in an early phase before soreness peaks.

Metabolomics is a comprehensive method to assess metabolites in tissue samples; extensive studies have been reported regarding metabolomic alterations by using various techniques, including nuclear magnetic resonance (Pechlivanis et al. 2013), gas chromatography/mass spectrometry (Major et al. 2006), and liquid chromatography-mass spectrometry (Yang et al. 2005). Among the developed techniques of metabolomics research, capillary electrophoresis time-of-flight mass spectrometry (CE-TOFMS) is a novel method for measuring metabolites of higher ionization and lower molecular weight and involves an easy preparation of samples (Kuwabara et al. 2013; Koike et al. 2014). Major advantages of CE-TOFMS include extremely high resolution, high throughput, and ability to simultaneously quantify all charged small molecule compounds in tissue samples virtually (Soga et al. 2003; Monto and Soga 2007). To date, no studies have been conducted to evaluate the application of CE-TOFMS to muscles after LC and/or MT.

Previous studies reported that massage effectively ameliorated deficient muscle contraction, an increase in muscle stiffness, inflammatory cell infiltration, and tissue injuries in an animal model of DOMS (Soga et al. 2003; Butterfield et al. 2008; Haas et al. 2012, 2013a,b). However, pain has not been studied in an animal model following LC and massage; therefore, the primary aim of this study was to establish a model by using rats to experimentally confirm the therapeutic effects of MT on mechanical hyperalgesia in DOMS. The second aim was to apply CE-TOFMS to the exercised rat muscle and to analyze the alteration of metabolites by MT in early phase before soreness peaked using previously reported animal model of DOMS (Taguchi et al. 2005; Fujii et al. 2008; Nasu et al. 2010), which would add new findings as one of objective effects of MT.

## Materials and Methods

### Animals

Male Sprague Dawley rats (SLC, Hamamatsu, Japan), weighing 160 g (6 weeks) at the beginning of the experiments, were used. Two or three animals were housed per laboratory cage under a 12-h light/dark cycle (lights on at 7:00 am) and controlled temperature (22 ± 1°C). Food and water were available *ad libitum* throughout the experiment. All experiments, including the housing of the animals, adhered to the Guideline for Care and Use of Laboratory Animals of the Institute of Laboratory Animal Resources, National Research Council (1996). Experimental procedures were approved by the ethical review board for animal experiments at the University of Toyama (Permit number; S-2010 MED-63 and A2013 MED-38).

### Grouping of the animals and experimental schedule

**Figure 1:**
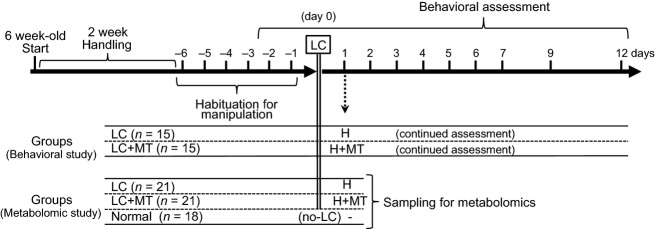
Time schedule of the experimental procedures. All animals were subjected to adequate handling before habituation for experimental manipulation, including manual therapy (MT), and holding of the trunk for behavioral assessment and withdrawal threshold measurement. Lengthening contraction (LC) was applied to the gastrocnemius muscle on day 0. After behavioral assessment on day 1, LC + MT group received handling (1 min) (H) before and after MT on the exercised muscles, while LC group received only handling (2 min). Three hours after MT (i.e. the next day after LC) and at the corresponding time, the gastrocnemius muscles were removed for metabolomics analyses. Normal group received no experimental manipulation.

Two experiments were performed (Fig.1): (1) behavioral study to establish MT in DOMS in the rat gastrocnemius and (2) metabolomic study to analyze metabolites in the gastrocnemius. Animals were randomly assigned to one of the following three groups: (1) Normal control group that received neither LC or MT (*n* = 18, metabolomic study), (2) LC group that received only LC, but not MT (*n* = 15, behavioral study; *n* = 21, metabolomic study), and (3) LC + MT group that received both LC and MT (*n* = 15, behavioral study; *n* = 21, metabolomic study). LC + MT group received handling (1 min) before and after MT, while LC group received only handling (2 min) (see below in detail). All animals were subjected to conventional animal handling for >10 min daily for 2 weeks until day −6 in order to reduce stress.

### Lengthening contraction procedures

To induce DOMS, LC was applied as reported previously (Taguchi et al. 2005). The left lower hind-leg muscle (i.e. gastrocnemius muscle) was used to evaluate the effects of MT, which is sufficiently large to identify and palpate a muscle belly from the skin by human fingers. Briefly, on day 0 (onset of LC), the animals were anesthetized with sodium pentobarbital (50 mg/kg, i.p.). LC was induced in the lateral head of the gastrocnemius muscle by electrical stimulation of the tibial nerve through a pair of needle electrodes inserted near the nerve. According to the previous studies for the rat DOMS model (Taguchi et al. 2005; Fujii et al. 2008; Nasu et al. 2010), electrical stimulation by a constant current stimulator [SEN-7203 (electronic stimulator) and SS-202J (a constant current stimulus isolator), Nihon Kohden, Japan] was applied for 1 s according to the following parameters: current strength of thrice the twitch threshold (<150 *μ*A) and a frequency of 50 Hz with pulse duration of 1 ms. The rat's paw and ankle joint were fixed to a foot plate. The foot plate was mechanically pulled to move the ankle joint from the plantar position (25° plantar-flexion) to dorsi-flexion (20°, total 45° range of motion) by using a linearized servomotor (CPL28T2B-06KD, Oriental Motor, Tokyo, Japan) for lengthening the gastrocnemius muscle during a 1 s period autonomously synchronizing with electrical stimulation (resulting in LC) and then returning to the starting position during a 3 sec resting period. This cycle was repeated 500 times. After recovering from anesthesia after LC, the behavior of the rats, including feeding, was normal.

### Withdrawal threshold measurement

A Randall–Selitto analgesiometer (Ugo Basile, Italy) equipped with a probe (tip diameter = 5 mm) was used to measure the withdrawal threshold in the deep tissues (i.e. gastrocnemius muscle). It has been previously shown that a probe with a tip diameter of 5 mm allows measurements of the muscle mechanical nociceptive threshold (Nasu et al. 2010). The animals were gently restrained around the trunk with a towel and socks to calm them during the experiments. The probe was applied to the lateral belly of the gastrocnemius muscle through shaved skin. Force was applied to the muscle through the probe at an increasing speed of 157 mN/s; the maximum applied force for loading was 2450 mN to avoid tissue damage. The pressure intensity at which an escape reaction occurred was defined as the withdrawal threshold. Withdrawal thresholds were measured seven times at intervals of 2–3 min, and the mean value of the latter five trials was considered the threshold value. Each experimental series was performed at approximately the same time (2:00 PM to 4:00 PM) to avoid circadian fluctuations. Training sessions before LC were performed for at least six consecutive days.

To determine if the changes in withdrawal thresholds, measured by using the Randall-Selitto analgesiometer, were not ascribed to cutaneous mechanical hyperalgesia, cutaneous mechanical hyperalgesia was measured by using self-made von Frey hairs (diameter: 0.5 mm, bending forces 36.5–1756.8 mN in quasi-logarithmic order) because mechanical strain induced by thin von Frey hairs can barely reach the deeper muscle layer (Takahashi and Mizumura 2004). The rats were restrained in the same way as the Randall–Selitto test, and each filament was applied thrice to the skin at intervals of 5–7 sec. The threshold was determined by using the limits method (by changing the direction of the forces applied to the von Frey hairs in an upward or downward direction). A positive response was defined when the animal showed at least two withdrawal responses. This procedure was performed prior to the Randall–Selitto test each day.

The all groups including the normal group received the behavioral assessments using the left leg for 6 days daily before LC to allow for habituation against the manipulation in (Fig.1). The experimenters were blind to the rat sample groups. The mean of the withdrawal thresholds during the last 2 days before LC was used as the baseline before LC.

### Manual therapy

**Figure 2:**
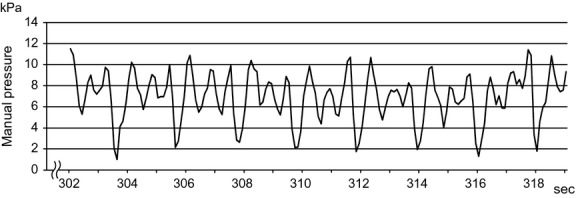
Example intermittent pressure data during manual therapy (MT). Mechanical pressures on the experimenter's thumb were monitored by using a strain gauge during MT to prevent the pressure from exceeding 12 kPa.

After assessing the withdrawal threshold on day 1 (following the LC day), the left gastrocnemius muscle of the rats in the LC + MT group received MT (manual massage) without anesthesia. MT consisted of the following three steps: (1) 1-min handling of the animals by their trunks to reduce stress and to enable relaxation, (2) 10-min application of intermittent and rhythmical compression (1–2 Hz) by using the experimenter's thumb, which was followed by (3) another 1-min handling of the animals to promote relaxation. Mechanical pressure on the thumb was monitored online through an interface by using a strain gauge [diameter = 5 mm (area of the sensor = 19.6 mm^2^); PS-10KC M3Z, PCD-331B-F, and DCS-100A, Kyowa Electronic Instruments, Tokyo, Japan] (Fig.2). The pressure was maintained at <12 kPa/19.6 mm^2^; total force over the whole area of the thumb was <2.8 N to avoid tissue damage and behavioral excitation. Animals in the LC group (non-MT) were handled only after assessing the withdrawal threshold.

The rats received MT thrice during the habituation period in order to be acclimated to the MT. Following habituation, animals became accustomed to the MT manipulation and did not show any stressful signs such as freezing, escaping from the hand, and fastening the claws. MT was performed during a fixed time during the day (3:00 PM to 5:00 PM), in order to avoid circadian fluctuations, and after the behavioral test, in order to prevent acute effects of MT on the pain threshold.

### Capillary electrophoresis time-of-flight mass spectrometry

The left lateral belly of the gastrocnemius muscle was removed after deep anesthesia and rapid decapitation 3 h after MT in the LC + MT group and at the same corresponding time the day after LC in the LC group. The tissue blocks were immediately frozen and transversely sectioned (thickness = 1 mm) with razor blades. The collected tissue samples were weighed (40–60 mg), transferred into micro tubes, and stored at −80°C until the assay. After the addition of 50% (v/v) acetonitrile solution (30 *μ*L/mg of muscle tissue) containing internal standards (20 *μ*mol/L methionine sulfone [MetSul] and 5 *μ*mol/L D-camphor-10-sulfonic acid [CSA] for cationic and anionic metabolites, respectively), the frozen muscle tissues were homogenized four times with a crusher (Shake Master NEO, BMS-M10N21 Biomedical Science, Tokyo, Japan) for 120 sec [shaking a 2.0 ml tube containing a metal ball (8.2 *g*) 25 times/sec]. Following centrifugation at 2300 *g* for 5 min at 4°C, three individually extracted tissue samples from the 400 *μ*L supernatant were mixed to obtain a single sample. Therefore, a single sample would include metabolites derived from three muscles of the same group (normal group, *n* = 6 samples from 18 individual muscles; LC group, *n* = 7 samples from 21 individual muscles; and LC + MT group, *n* = 7 samples from 21 individual muscles). The sample solution was ultrafiltrated by using the Ultrafree-MC PLHCC, Centrifugal Filter Device (Human Metabolome Technologies, Tsuruoka, Japan) with a 5-kDa (molecular weight) cutoff to remove proteins. The filtrate was evaporated, dissolved in 50 *μ*L of ultra-pure water, and then analyzed by using CE-TOFMS.

CE-TOFMS measurements were performed by using an Agilent Capillary Electrophoresis System equipped with an Agilent 6210 Time of Flight mass spectrometer (Agilent Technologies, Waldbronn, Germany). Cationic metabolites were analyzed with a fused silica capillary (50 *μ*m, inner diameter × 80 cm, total length) with a commercial cation electrophoresis buffer (Solution H3301-1001; Human Metabolome Technologies) as the electrolyte. The sample was injected at a pressure of 50 mbar for 10 sec (approximately 10 nL). The applied voltage was set at 27 kV. Electrospray ionization-mass spectrometry was conducted in the positive ion mode, and the capillary voltage was set at 4 kV. The spectrometer was scanned from 50 to 1000 m/z (mass-to-charge ratio). Other conditions were similar to those mentioned in a previous analysis (Soga et al. 2003).

Anionic metabolites were also analyzed with a fused silica capillary with a commercial anion electrophoresis buffer (Solution H3302-1021; Human Metabolome Technologies). The sample was injected at a pressure of 50 mbar for 25 sec (approximately 25 nL). The applied voltage was set at 30 kV. Electrospray ionization-mass spectrometry was conducted in the negative ion mode, and the capillary voltage was set at 3.5 kV. The spectrometer was scanned from 50 to 1000 m/z. Other conditions were similar to the cation analysis.

### Analysis of capillary electrophoresis time-of-flight mass spectrometry data

Raw data obtained via CE-TOFMS were processed with analysis software (MasterHands, Human Metabolome Technologies). Signal peaks corresponding to isotopomers, adduct ions, and other product ions of known metabolites were excluded; other signal peaks potentially corresponding to authentic compounds were extracted and their migration time was normalized by using those of the internal standards. Thereafter, the peaks were aligned across the samples according to the m/z and normalized migration time values. Finally, peak areas were normalized against those of the internal standards (MetSul and CSA for cations and anions respectively). The relative area values obtained were further normalized by the amounts of samples. Annotation lists were produced from the CE-TOFMS measurements of standard compounds, and they were aligned with the datasets according to similar m/z values and normalized migration time values; a difference of ± 10 ppm for the m/z values and ± 0.5 min for the migration time were permitted.

In addition, the analyses in the present study enabled measurement of the absolute quantities of the predetermined 110 major metabolites based on the peak area of their internal controls. The quantity of these 110 metabolites was reliably analyzed and compared across different experimental batches; therefore, we were able to quantify the absolute concentrations of these metabolites.

### Statistical analysis

All values were expressed as mean values ± standard error of means. All statistical analyses were performed by using the software package SPSS (v. 19; IBM, Armonk, NY). Percentage changes in withdrawal thresholds were examined with the two-way repeated measures by using analysis of variance (ANOVA) with the group and day as factors followed by Bonferroni post hoc comparison tests. Relative and absolute amounts of muscle metabolites in the three different groups (i.e. normal, LC, and LC + MT) were examined with one-way ANOVA followed by LSD post hoc comparison tests. Differences were considered statistically significant at *P *<* *0.05.

## Results

### Effects of manual therapy

**Figure 3:**
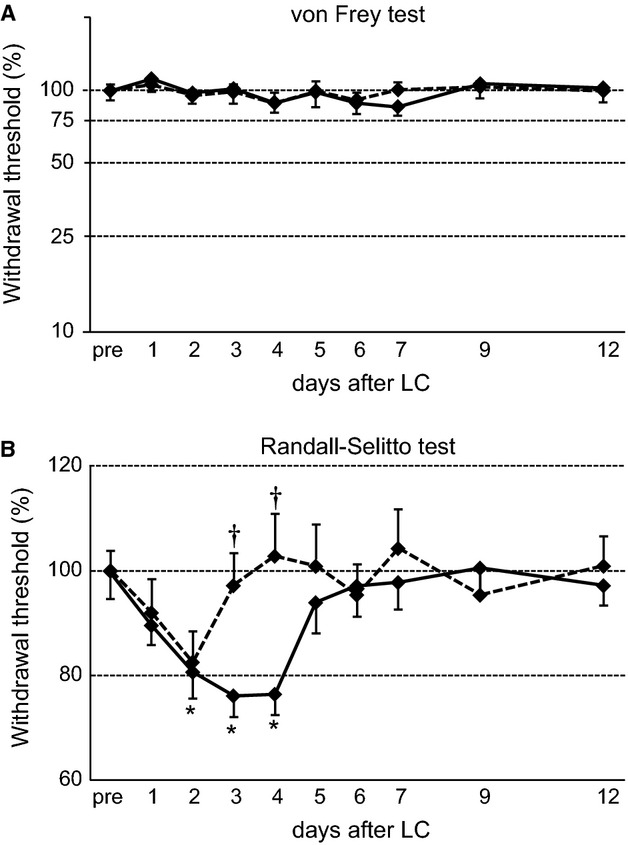
Time course of mechanical withdrawal thresholds measured by using von Frey hairs (A) and the Randall-Selitto method (B). A*,* The cutaneous mechanical withdrawal threshold did not change after LC and MT. Ordinate, threshold expressed as percentages of the baseline threshold before LC (pre) in a logarithmic scale. B, The rats in the LC group (solid line) showed significant decreases in the mechanical thresholds after LC compared to the pre-LC group (*P <* *0.05, Bonferroni post-hoc test). However, the rats in the LC + MT group (dotted line) showed an increase in the mechanical thresholds compared to the LC group (^†^*P *<* *0.05, Bonferroni post-hoc test). Ordinate, threshold expressed as percentages of the baseline threshold before LC (pre) in a linear scale.

The withdrawal thresholds of the rats with or without MT after LC were assessed daily to analyze the effects of MT on DOMS of the rat gastrocnemius muscle (Fig.3). In the von Fly filament test, there were no significant main effects of day (*F*_9,252_ = 1.40, *P *>* *0.05) and group (*F*_1,28_ = 0.00, *P *>* *0.05) or significant interactions between the group and day (*F*_9,252_ = 0.31, *P *>* *0.05; Fig.3A). However, there were significant main effect of day and significant interaction between group and day in the Randall–Selitto test (*F*_9,252_ = 7.12, *P *<* *0.001; *F*_9,252_ = 4.20, *P *<* *0.001; Fig.3B). In the post hoc comparisons, LC significantly decreased the withdrawal threshold 2–4 days after LC compared to the baseline threshold before LC in the LC group (*P *<* *0.05–0.005, Fig.3B). According to these results, LC successfully induced DOMS in the rat gastrocnemius muscle without cutaneous hyperalgesia.

Significant differences between groups were also observed with the post-hoc comparisons. Following the application of MT, there were no significant differences between the groups on days 1 and 2 after LC (*P *>* *0.05, Fig.3B). However, the withdrawal threshold rapidly returned to the baseline level in the LC + MT group, and there were significant differences in the withdrawal thresholds between groups on days 3 and 4 following MT (*P *<* *0.01, Fig.3B). Therefore, MT significantly ameliorated DOMS in the gastrocnemius muscle.

### Alteration of metabolites following lengthening contraction and manual therapy

The metabolism of skeletal muscle has been reported to change by effleurage within a few hours during the early phase after MT (Crane et al. 2012). Therefore, we analyzed muscle metabolisms 3 h after MT (i.e. 1 day after LC). A total of 171 metabolites (111 for cation and 60 for anion modes) were detected via CE-TOFMS analysis in the 20 gastrocnemius muscle samples (*n* = 6, normal group; 7, LC group; and 7, LC + MT groups). Metabolites that contained data below the detection limit in >2 samples were discarded; 114 metabolites (82 for cation and 32 for anion modes) were examined in total.

**Figure 4:**
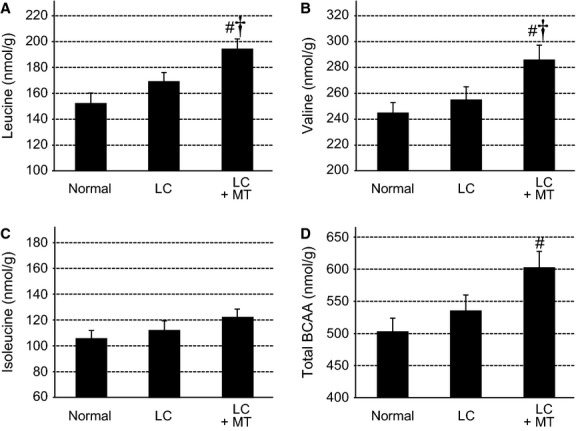
Metabolomics analysis of branched-chain amino acids (BCAAs) including leucine (A), valine (B), isoleucine (C), and total BCAA (D) in the rat gastrocnemius. Application of MT increased concentrations of leucine (A), valine (B), and total BCAA (D), but not of isoleucine (C). ^#^significantly different between Normal and LC + MT (*P *<* *0.05); ^†^significantly different between LC and LC + MT (*P *<* *0.05, LSD post hoc test following two-way repeated measures by using analysis of variance)

Of the detected metabolites, 19 metabolites showed significant differences among the groups (Fig.4 and Tables1 and 2). The remaining detected 95 metabolites (i.e. adenosine, ADP, ATP, creatine, creatinine, glucose 1-phosphate, glucose 6-phosphate, GTP, and lactic acid) did not show any significant differences even though the gastrocnemius muscles of the rats underwent LC and MT. Some of the 19 metabolites that had significant differences were analyzed in absolute concentrations (Fig.4 and Table1; see Materials and Methods). The significant differences in the metabolites were grouped into three categories: (1) differences between the LC and LC + MT groups (eight metabolites indicated by † in Fig.4 and Tables1 and 2), which reflected the effects of MT after LC, (2) differences between the normal and LC + MT groups (15 metabolites indicated by # in Fig.4, and Tables1 and 2), which reflected combined effects of LC and MT compared with the normal control, and (3) differences between the normal and LC groups (13 metabolites indicated by * in Tables1 and 2), which reflected simple effects of LC on day 1.

**Table 1:** Comparison of metabolite concentrations among the three experimental groups.

Metabolites	Related metabolic pathway	Normal (*n* = 6)		LC (*n* = 7)		LC + MT (*n* = 7)	
		Mean (SE) (nmol/g)	%	Mean (SE) (nmol/g)	%	Mean (SE) (nmol/g)	%
Phenylalanine	Aromatic amino acid metabolism	63.1 (2.3)	100	69.0 (2.2)	109	76.8#^,^† (2.9)	122
Histidine	Urea cycle	203.3 (13.4)	100	169.3* (5.9)	83	163.4# (7.0)	80
Homoserine	Essential amino acid metabolism	2.9 (0.1)	100	2.1* (0.2)	73	2.8† (0.2)	99
Malic acid	TCA cycle	247.9 (22.9)	100	238.9 (6.0)	96	193.4#^,^† (10.9)	78
NAD+	Nicotinamide metabolism/energy carriers	404.7 (21.4)	100	320.8* (32.3)	79	303.4# (20.6)	75
Succinic acid	TCA cycle	79.5 (3.7)	100	63.7* (3.5)	80	67.5# (1.8)	85
CTP	Pyrimidine metabolism	42.9 (1.8)	100	55.5* (2.3)	129	57.5# (1.8)	134
Ribulose 5-phosphate	Pentose phosphate pathway	31.1 (3.7)	100	52.9* (8.1)	170	54.0# (3.4)	174
GABA	Urea cycle/TCA cycle	6.8 (0.6)	100	5.1* (0.3)	75	4.5# (0.2)	66
Spermidine	Polyamine metabolism	1.6 (0.4)	100	2.7 (0.2)	168	3.6# (0.5)	222
Ribose 5-phosphate	Pentose phosphate pathway/nicotinamide metabolism	11.0 (1.5)	100	16.5* (1.5)	150	13.5 (0.9)	123

^*^Significant difference (*P* < 0.05) between Normal and LC; ^#^Significant difference (*P* < 0.05) between Normal and LC + MT.

†Significant difference (*P* < 0.05) from LC.

**Table 2:** Comparison of relative amounts of metabolites (relative areas) among the three experimental groups.

Metabolites	Related metabolic pathway	Normal (*n* = 6)		LC (*n* = 7)		LC + MT (*n* = 7)	
		Mean (SE) × 10^−3^ (relative area)	%	Mean (SE) × 10^−3^ (relative area)	%	Mean (SE) × 10^−3^ (relative area)	%
N-Methylalanine	Pyruvic acid metabolism	1.39 (0.08)	100	1.02* (0.04)	74	1.24† (0.07)	89
3-Guanidinopropionic acid	(Creatine analog)	2.82 (0.09)	100	2.81 (0.13)	100	2.30#^,^† (0.10)	82
Carnitine	Fatty acid metabolism	228 (7.79)	100	233* (3.88)	102	251† (5.94)	110
Ethanolamine phosphate	Phospholipid metabolism	3.09 (0.16)	100	4.30* (0.18)	139	5.18# (0.45)	168
UDP-glucose UDP-galactose	Polysaccharide/glycometabolism	1.85 (0.11)	100	2.18* (0.10)	117	2.22# (0.12)	119
Kynurenine	Aromatic amino acid metabolism	1.18 (0.43)	100	0.29* (0.02)	24	0.27# (0.02)	23

^*^Significant difference (*P* < 0.05) between Normal and LC; ^#^Significant difference (*P* < 0.05) between Normal and LC + MT.

†Significant difference (*P* < 0.05) from LC.

Of the metabolites with significant differences between the LC and LC + MT groups, three metabolites were proteinogenic amino acids, including leucine (Leu), valine (Val) (Fig.4) and phenylalanine (Phe) (Table1). Leu, Val, and isoleucine (Ile) constitute the branched-chain amino acids (BCAAs: amino acids with aliphatic side-chains and a branch). The absolute individual concentrations of these 3 BCAAs (A, B, C) and the total concentration of these 3 BCAAs (D) in the samples are shown in Figure4. The Leu concentration was significantly different among groups (*F*_2,17_ = 7.55, *P *<* *0.005), and MT significantly increased the concentration of Leu in the LC + MT group compared to both the normal controls (*P *<* *0.005) and the LC group (*P *<* *0.05; Fig.4A). Similarly, the Val concentration was different among groups (*F*_2,17_ = 4.43, *P *<* *0.05; Fig.4B), and MT significantly increased the Val concentration in the LC + MT group compared to the normal (*P *<* *0.05) and LC (*P *<* *0.05) groups. The Ile concentration was not significantly different among groups (*F*_2,17_ = 1.45, *P *>* *0.1; Fig.4C). However, the total concentration of the BCAAs (Leu + Val + Ile) differed significantly among groups (*F*_2,17_ = 4.68, *P *<* *0.05). Furthermore, MT significantly increased the total concentration of the BCAAs in the LC + MT group compared to the normal group (*P *<* *0.05) and tended to increase total BCAA concentration compared to the LC group (*P *=* *0.056; Fig.4D).

## Discussion

In the present study, MT ameliorated LC-induced hyperalgesia (DOMS) in the rat gastrocnemius muscle. It was revealed for the first time that the concentrations of the several metabolites in the muscle were altered following LC and MT, according to the metabolome analysis using CE-TOFMS.

### Significance of an animal model for manual therapy

In the present study, we established an animal model (i.e. rats) to assess effects of muscle compression on mechanical hyperalgesia in DOMS. It has been reported that hyperalgesic muscles in DOMS are similar to clinical trigger points, which suggests that DOMS can be used as a model for studying the mechanisms of trigger points (Mizumura et al. 2010). Consistent with this idea, repetitive eccentric exercise induced trigger points in human subjects (Itoh et al. 2004). Trigger points are suggested to be responsible for musculoskeletal pain (Simons et al. 1999); compression at trigger points has also been effective for musculoskeletal pain, such as chronic lower back, neck, shoulder, and knee pain and fibromyalgia (Hains and Hains 2000, 2010; Hains 2002a,b). Based on the present results, manual compression at trigger points might also be effective for acute musculoskeletal pain. Consistent with this idea, it has been previously reported that massage in acute phase, including compression, at trigger points ameliorated DOMS and neck pain (Nelson 2013; Takamoto et al. 2013). Further studies are required to investigate whether MT on trigger points might induce metabolite alternations similar to those in the present study.

### Therapeutic role of branched-chain amino acids

According to the metabolome analyses using CE-TOFMS, the concentrations of several metabolites were different after LC and/or MT. It was observed that two essential amino acids, Leu and Val of the BCAAs, increased immediately (3 h) after MT (Fig.4). BCAAs are abundant and catabolized in the skeletal muscle, inhibit protein decomposition, and enhance protein synthesis. BCAAs have been reported to ameliorate DOMS and muscle damage in DOMS (Shimomura et al. 2006; Greer et al. 2007; Jackman et al. 2010; Ra et al. 2013). The complete catabolic pathways for BCAAs in the muscle are located in the mitochondria; BCAAs are transaminated by branched-chain aminotransferase to *α*-ketoisocaproate, decarboxylated to isovaleryl-CoA by the mitochondrial enzyme branched-chain *α*-ketoacid dehydrogenase (BCKDH), and finally converted to Acetyl-CoA derivatives for entering the citric acid (TCA) cycle. The latter reaction via BCKDH is the rate-limiting step of BCAA catabolism. The BCAA catabolism is reported to be regulated by exercise; endurance exercise activates the BCKDH complex in humans (Wagenmakers et al. 1989) and rat skeletal muscles (Shimomura et al. 1993, 1995). These findings suggest that BCAAs as energy sources are catabolized following exercise, and they expand the pool of TCA cycle intermediates and gluconeogenesis (Shimomura et al. 2004).

Leu is catabolized as an energy source (see above) and is reported to increase muscle protein synthesis and inhibit protein degradation (Mordier et al. 2000; Bolster et al. 2004). Furthermore, *in vitro* treatment of skeletal muscles with Leu significantly increased the expression of peroxisome proliferator-activated receptor coactivator 1 alpha (PGC-1*α*; an important stimulator of mitochondrial biosynthesis), mitochondrial contents, and oxidative metabolism (Handschin et al. 2007). In human muscle biopsies, MT induced PGC-1*α* translocation into the nucleus to increase the expression of COX7B and ND1 mRNA, which is involved in mitochondrial respiration (Crane et al. 2012). Based on these findings, Leu is required for muscles during work and recovery from LC. We demonstrated that the application of MT after LC increased Leu concentrations, which suggests that the effects of MT are mediated partly through BCAAs, including Leu, and might enhance mitochondrial biogenesis and energy metabolism.

### Other metabolites

In this present study, we demonstrated that MT increased carnitine concentrations in the muscle. Carnitine, one of the most popular commercial supplements, has been widely accepted as a potential ergogenic acid because of its important role in the conversion of fat into energy (Cerretelli and Marconi 1990). Carnitine promotes carnitine-dependent transport of fatty acids into the mitochondrial membrane, which is a rate-limiting step in long chain fatty acid oxidation (McGarry and Brown 1997). It has been reported that carnitine supplementation enhances fatty acid oxidation during exercise (Gorostiaga et al. 1989). Therefore, the increase of carnitine after MT might transport fatty acids into the mitochondrial matrix and increase energy production in the TCA cycle.

Malic acid, NAD+, and succinic acid, which decreased after LC and/or MT, are TCA cycle substrates or metabolic byproducts. Among these metabolites, only malic acid specifically decreased after MT, while NAD+ and succinic acid decreased after LC. It has been previously reported that the concentration of these metabolites increased during exercise, possibly through an anaplerotic pathway (Gibala et al. 2000). It was observed that the samples were collected 1 day after LC and the reversed changes in these metabolites after LC and MT might reflect recovery processes from the acute metabolic changes after LC. However, it remains unclear whether these changes contributed to the amelioration of DOMS. Further studies are required to investigate physiological roles of these metabolites in pain sensation.

### Unchanged metabolites

In general muscle physiology, the bioenergetic provision for exercise is divided into three phases: (1) high energy phosphate system supplied by phosphocreatine and stored as ATP, (2) anaerobic glycolytic system supplied by stored glycogen and blood glucose, and (3) aerobic oxidative system supplied by glycogen, glucose, fat, and proteins. Time for these energy provision systems to recover by half is reported to be <20 min (Wells et al. 2009). In human studies, it was reported that concentrations of muscle metabolites (i.e. ATP, phosphocreatine, and lactate) transiently changed immediately after exercise, but rapidly restored within the several minutes (Bogdanis et al. 1995; Dawson et al. 1997). In the present study, muscle samples were collected 1 day after exercise and 3 h after the application of MT. This sampling timing might result in the recovery of most detected metabolites (95/114 metabolites, including ATP and creatinine), and these metabolites might not be related to DOMS or the ameliorative effects of MT.

## Conclusions

In this study, MT significantly ameliorated DOMS, which was induced via LC in the rat gastrocnemius. It has been reported that LC induces the overextension of sarcomeres, which might further induce membrane damages and local contracture in the exercised muscle (Ge et al. 2011; Bron and Dommerholt 2012). Local contracture might increase mitochondrial respiration in the muscle (Bron and Dommerholt 2012), which induces alteration of metabolite profiles as shown in the present study. The ionic changes associated with metabolite changes due to LC might stimulate specific ion channels expressed in the dorsal root ganglion cells (Fujii et al. 2008). MT might normalize sarcomere disorganization (Haas et al. 2012), which might reduce contracture and metabolite changes. The present results are consistent with the following hypothesis: metabolic alterations caused by MT might be involved in physiological mechanisms of therapeutic effects on DOMS. This was the first study, to the best of our knowledge, to report that MT significantly reduced mechanical hyperalgesia in DOMS and altered metabolites in the rat muscle. Further studies are required to prove or disprove this hypothesis, and also to analyze how alteration of other factors such as inflammatory mediators by MT affects mechanical hyperalgesia in this animal model of DOMS.

## Conflict of Interest

The authors report no conflict of interest.
